# Vaginal group B *Streptococcus* colonization among pregnant women in Ethiopia: Prevalence, risk factors, and antimicrobial susceptibility

**DOI:** 10.1016/j.isci.2025.113140

**Published:** 2025-07-17

**Authors:** Abeba Mengist

**Affiliations:** 1 Department of Medical Laboratory Science, College of Medical and Health Sciences, Debre Markos University, Debre Markos, Ethiopia

**Keywords:** medical microbiology, women’s health

## Abstract

Group B *Streptococcus* (GBS) colonization during pregnancy poses significant public health risks, contributing to adverse outcomes like neonatal septicemia, meningitis, and pneumonia. Transmission from mother to newborn during delivery is common. In Jimma, Ethiopia, limited data exist on GBS prevalence, risk factors, and antimicrobial resistance among pregnant women, with no universal screening protocol to mitigate neonatal infections.

This study assessed GBS prevalence, antimicrobial susceptibility, and associated risk factors among 200 pregnant women at Jimma University Hospital. Vaginal swabs collected at 35–37 weeks gestation were cultured and tested for GBS using standard methods.

Results showed a colonization rate of 13% (26/200), higher in multigravida (17%) compared to primigravida (3.4%). Resistance rates to ampicillin, penicillin, clindamycin, and erythromycin were 30.8%, 23.1%, 19.2%, and 15.4%, respectively.

The findings underscore the importance of routine screening and susceptibility testing to inform prophylactic strategies and curb neonatal infections.

## Highlights

• Vaginal GBS colonization in Jimma, Ethiopia was 13% (26 of 200 women tested)
• GBS more common in multigravida (17%) than in primigravida women (3.4%)
• GBS isolates showed resistance to ampicillin, penicillin, and clindamycin
• Screening and susceptibility testing for GBS is key to proper prophylaxis

## Graphical abstract

**Figure:**
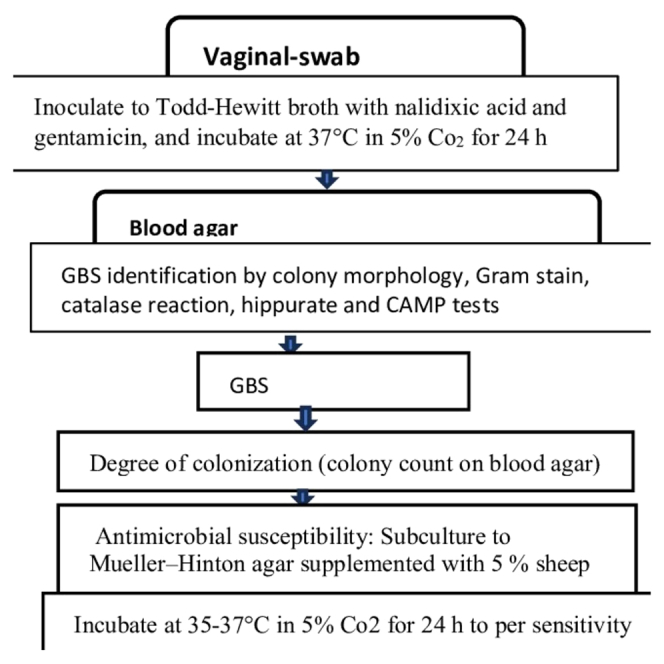

## Introduction

*Streptococcus agalactiae*, also known as Group B *Streptococcus* (GBS) is the normal flora of the female urogenital tract and rectum. The importance of GBS is that it can be transferred to a neonate from their mother’s genital tract during delivery and cause sepsis and meningitis in newborns. GBS has been associated with adverse pregnancy outcomes, such as low birth weight, pre-term delivery, and prelabor rupture of the membranes.^1^ Even, in the absence of maternal chemoprophylaxis, up to 50% of neonates born to colonized women acquire GBS colonization and 1%–2% of these neonates acquire invasive disease.^2^

GBS neonatal infection could be early-onset GBS disease (EOGBSD), which occurs within the first week of life, and late-onset GBS disease (LOGBSD), which occurs between one week to 3 months of age.^3^^,^^4^ EOD is associated with high mortality of infants (5%–6% among mature infants and up to 25% among premature newborns).^5^ Infants who have such infections may require prolonged hospitalization, and 25%–50% of those survivors may have mental retardation or visual loss.^6^

Pregnant women who are GBS carriers are also at risk for severe infections, such as maternal pelvic infection, puerperal infection, and urinary tract infection.^7^ Puerperal infections and complicated abortion are some of the leading causes of maternal death in Ethiopia, which could be associated with *S. agalactiae* colonization.^8^

There is a large geographical variation in GBS colonization rates with about 15%–40% of pregnant women colonized by GBS in different regions of the world.^9^ Studies also indicated a variation of maternal GBS colonization by population characteristics, such as age, parity, socio-economic status, geographic location, the presence of sexually transmitted diseases, and sexual behavior. Variable rates of GBS colonization (7.2%–20.6%) among pregnant women were also reported from different parts of Ethiopia.^10^^,^^11^^,^^12^^,^^13^

GBS is found in different sites of the healthy adults, but investigators have paid close attention to its vaginal colonization. In the absence of a licensed GBS vaccine, universal screening of mothers for vaginal or rectal GBS colonization at 35–37 weeks of gestation and selective intrapartum antibiotic prophylaxis (IAP) for all screen-positive women is the strategy currently recommended to reduce the incidence of colonization in neonates and prevent EOGBSD.^14^ In the United States, since the early 1990s, medical interventions have resulted in an 80% decline in the incidence of EOGBSD from 1.7 to less than 0.4 cases per 1,000 live births in recent years.^14^

Penicillin is the first-line agent for the treatment of GBS infections, while ampicillin is considered as an acceptable alternative. In penicillin-allergic women, who are not at high risk for anaphylaxis, cefazolin is considered the agent of choice for intrapartum prophylaxis because of its narrow spectrum of activity and ability to achieve high intra-amniotic concentrations.^14^ Otherwise, vancomycin is recommended for those with a history of anaphylaxis and when GBS is resistant to erythromycin and clindamycin. If the GBS is sensitive to one of these antibiotics, either erythromycin or clindamycin is recommended for intrapartum chemoprophylaxis.^14^ There are concerns about the emergence of drug resistance in parts of the world where IAP is practiced.^15^^,^^16^^,^^17^ Updated information on the trends of drug resistance on commonly used drugs is needed for practicing professionals in the study’s location.

Until today, there are no screening strategies in Ethiopia to reduce the burden of GBS neonatal infection. This study, aimed at determining the vaginal carriage rate of *S. agalactiae* among pregnant women and antimicrobial susceptibility pattern of isolates to reduce transmission of *S. agalactiae* from colonized women to their neonates. An attempt was also made to identify possible risk factors in order to generate local data that will inform the development of rational interventions for GBS infection and disease.

## Results

### Socio-demographic data

A total of 200 pregnant women were included in this study. The age of the women ranged from 16 to 40 years with a mean age of 26.3 years (SD ±5). As shown in Table 1, the majority (73.3%) of the participants was between the ages of 21–30 years. With respect to the educational status, 45% were who had only attended primary school followed by secondary (21.5%), post-secondary (18.5%), and illiterate (15%). With respect to the dwelling area, 79% reported living in an urban area and 21% in a rural area. Most study participants were housewives (66.5%) and the rest were individuals who employed in the governmental or non-governmental organization, merchants, and students.

**Table 1:** Socio-demographic characteristics of pregnant women (*N* = 200) investigated for GBS

Socio-demographic characteristics	Frequency	Percent (%)
Age in years		
16–20	20	10
21–25	81	40.5
26–30	66	33
31–35	23	11.5
36–40	10	5
40+	0	0
Residence		
Urban	158	79
Rural	42	21
Educational level		
Illiterate	30	15
Primary	90	45
Secondary	43	21.5
Post-secondary	37	18.5
Occupation		
House wife	133	66.5
Employed	54	27
Merchant	3	1.5
Student	10	5
Total	200	100

### GBS colonization rate and risk factors

Among the 200 pregnant women screened, 26 (13%) were colonized with GBS (Table 2). Of the 26 (13%) pregnant women colonized by GBS, 7 (26.9%) had light infections while 19 (73.1%) were highly colonized (Table 3). No significant association was found between maternal age, residence area, education, occupation, and GBS colonization rate (*p* > 0.05) (Table 4). Furthermore, with respect to the previous obstetric history (i.e., history of stillbirth, preterm labor, and spontaneous abortion), none of the preterm women were colonized by GBS. However, the relationship between GBS colonization and previous obstetric history was not statistically significant (*p* > 0.05) (Table 4). In contrast, gravida was significantly associated with higher GBS colonization rates as shown in Table 4. Colonization rates among multigravida 24/141 (17%) women were significantly higher compared to primigravid women 2/59 (3.4%) (*p* < 0.05) (Table 4).

**Table 2:** Isolation and number of GBS carriers (*N* = 26) among pregnant women investigated for GBS

Pregnant women	Number	Percent (%)
No. of GBS carriers	26	13
GBS not isolated	174	87
Total	200	100

**Table 3:** Degree of colonization in vaginal samples of GBS carriers

Degree of colonization	Pregnant women	
	Number	%
+1	4	15.4
+2	3	11.5
+3	8	30.8
+4	11	42.3
Total	26	100

**Table 4:** Association between socio-demographic, obstetric factors, and GBS colonization among pregnant women (*n* = 200) investigated for GBS

Variables	Colonization		Total	*p*-value
	Colonized, No (%)	Non-colonized, No (%)		
Age in years				
16–20	2 (10)	18 (90)	20	
21–25	14 (17.3)	67 (82.7)	81	
26–30	6 (9.1)	60 (90.9)	66	
31–35	2 (8.7)	21 (91.3)	23	
36–40	2 (20)	8 (80)	10	0.529
Residence				
Urban	20 (12.7)	138 (87.3)	158	
Rural	6 (14.3)	36 (85.7)	42	0.780
Educational level				
Illiterate	6 (20)	24 (80)	30	
Primary	14 (15.6)	76 (84.4)	90	0.238
Secondary	4 (9.3)	39 (90.7)	43	
Post-secondary	2 (5.4)	35 (94.6)	37	
Occupation				
House wife	22 (16.5)	111 (83.5)	133	
Employed	2 (3.7)	52 (96.3)	54	0.090
Merchant	0 (0)	3 (100)	3	
Student	2 (20)	8 (80)	10	
Gravidity				
Primigravida	2 (3.4)	57 (96.6)	59	
Multigravida	24 (17)	117 (83)	141	0.009
Previous obstetric history				
Normal healthy baby	19 (11.7)	144 (88.3)	163	
Preterm	0 (0)	10 (100)	10	0.057
Spontaneous abortion	4 (36.4)	7 (63.6)	11	
Stillbirth	3 (18.8)	13 (81.2)	16	

### Antimicrobial susceptibility pattern of GBS

The susceptibility patterns of GBS (*n* = 26) isolated from pregnant women against four antimicrobial agents is presented in Table 5. Most GBS isolate, 84.6% to erythromycin and 80.8% to clindamycin were susceptible. Relatively, GBS showed high resistance to penicillin G and ampicillin with 23.1% and 30.8%, respectively.

**Table 5:** Antimicrobial susceptibility pattern of GBS (*n* = 26) isolated from pregnant women

Antimicrobials	Susceptible, No (%)	Intermediate, No (%)	Resistant, No (%)
Penicillin G	20 (76.9)	–	6 (23.1)
Ampicillin	18 (69.2)	–	8 (30.8)
Erythromycin	22 (84.6)	–	4 (15.4)
Clindamycin	21 (80.8)	–	5 (19.2%)

## Discussion

GBS is a leading cause of sepsis and meningitis in neonates with a high case fatality rate of about 40%–80% in developed countries, yet the magnitude of this infection in developing countries such as Ethiopia has not been adequately studied.^18^

GBS is found in different sites of the healthy adults, but investigators have paid close attention to its vaginal colonization.^7^ Vertical transmission of GBS affects neonates as the most frequent infection responsible for sepsis in developing and developed nations. Although screening and prophylactic treatments have helped decrease mortality rates to 5%, a clear estimation of disease burden in many developing countries including Ethiopia remains unrecognized.^19^

This study showed that 13% of pregnant women who attended antenatal cares follow up in the study area had vaginal colonization with *S. agalactiae.* A comparable rate of colonization with the current study was reported in some parts of Ethiopia among pregnant women, which report, 7.2%–9%.^8^^,^^11^^,^^20^ The previous study was done by Mengist et al. in the same study area and showed higher colonization rate (19%) compared to the present study.^10^ This difference might be because of the site of sample collection (vaginal vs. vaginal and rectal sample). The Centers for Disease Control (CDC) have also recommended swabbing both the vagina and rectum increases the yield substantially compared with sampling either the vagina or the rectum alone.^14^ A higher proportion of pregnant women (73.1%) in the present study had heavy colonization, and it is reported that the likelihood of neonatal colonization at birth is higher if the mother is heavily colonized.^21^

Higher GBS colonization rates (31%–32.6%) were reported from Trinidad,^22^ Zimbabwe,^23^ and Brazil^24^ and lower colonization rates (1.8%–2.33%) from Mozambique^25^ and South India^26^ as compared to the current study. These differences indicate significant country variations. The rate of GBS isolation is different and depends on selected culture media, the number of sampled areas, and probably socioeconomic condition and geographic location. Race and geographical area may have an effect on the prevalence of GBS colonization due to differences in nutrition, socioeconomic status, sexually transmitted diseases, or host immunity.^10^

Even though knowledge about risk factors contributing for GBS colonization in pregnant women is relevant to minimize the morbidity, mortality associated with maternal and neonatal GBS infections,^10^ in the present study, the GBS colonized and non-colonized women were quite similar with respect to sociodemographic and previous obstetric history. These observations are in contrast to findings in some previous studies, in which age^27^ and previous history of stillbirth and spontaneous abortion^28^ have been associated with GBS colonization.

Multigravida was associated with an increased risk of colonization, which is consistent with the findings of other investigators.^24^^,^^29^ Since the rates and risk factors of maternal GBS colonization may vary in different communities and need to be evaluated in each country to allow the most appropriate preventive strategy to be selected. Although GBS screening is not the standard of care in Ethiopia, to select the most appropriate strategy (i.e., either the universal prenatal screening or risk-based approach as recommended by the CDC),^14^ the prevalence of GBS in the population has to be investigated.

In the present study, the resistance pattern of GBS to penicillin and ampicillin was 23.1% and 30.8% respectively. This is in contrast with the CDC recommendation, which did not find any resistance to penicillin.^14^ These findings were comparable to those reported in another study.^30^ A study conducted in Nekemete, Ethiopia also demonstrated resistance of penicillin was 77.3%.^11^ The resistance of GBS isolates against penicillin G and ampicillin in the present study is in contrary from a study done in different studies where all isolates were sensitive to penicillin and ampicillin.^10^^,^^12^ This may be due to the wide and nonprescription use of beta-lactam antimicrobials in our community because of a weak drug control mechanism. However, the finding has significant clinical implication regarding the use of beta-lactam antimicrobials in the treatment of GBS related infections both in mothers and their neonates. Therefore, to reduce penicillin resistance in GBS infections, it is essential to strengthen antibiotic stewardship programs, enhance drug regulation, and educate healthcare providers and the public about responsible antibiotic use. Additionally, establishing surveillance systems and promoting research into alternative therapies can help combat resistance effectively.

In our study, 15.4% of the isolates were resistant to erythromycin and 19.2% to clindamycin. These results are not very different from those found in the literature, in which resistance rates of 20.5%–22.6% have been reported to erythromycin,^12^^,^^29^^,^^31^ while, resistance to clindamycin was 17.9%–18.2%.^11^^,^^24^ Our rate of resistance to erythromycin and clindamycin is lower than rates in Birmingham (erythromycin, 39.5% and 26.4% to clindamycin)^30^ and in Nigeria (40% to erythromycin).^32^ Varying results of GBS susceptibility to antibiotics has been reported which might be due to the differences in antimicrobial use, prophylaxis practice, and serotype frequency may result in regional differences in the susceptibility of GBS to antibiotics.^33^ Since clindamycin and erythromycin are another alternative antibiotics recommended by the CDC for pregnant women who are allergic to penicillin, the resistance level underlines the need of carrying out a susceptibility test.

In conclusion, the prevalence of vaginal GBS in the present study is comparable to that reported elsewhere. GBS colonization was not affected by any of the socio-demographic and obstetric characteristics but has a significant association with multigravidity. Finally, the author concludes that screening for GBS among pregnant women is crucial, as GBS screening during pregnancy is not yet standard care in Ethiopia. Intrapartum antimicrobial prophylaxis should then be provided to women found to be colonized, which will prevent the transfer of GBS during delivery to neonates and avoid the development of early-onset GBS syndrome. The resistance to penicillin is notably high. Thus, initiatives should be taken to reduce the risk of multiple drug resistance.

### Limitations of the study

The design of the study did not attempt to use latex agglutination tests for confirmation of GBS due to budget and laboratory resource constraints. In addition, serotyping of GBS was not performed due to lack of group specific antisera.

## Resource availability

### Lead contact

Further information and requests for resources and reagents should be directed to and will be fulfilled by the lead contact, Abeba Mengist (abymaa@gmail.com).

### Materials availability

The study did not generate new materials.

### Data and code availability

• All the data used to support the findings of this are included within the article.
• The code related to this article can be accessed by reaching out to the lead contact, provided the request is reasonable.
• Any additional information required to reanalyze the data reported in this paper is available from the lead contact upon request.

## Acknowledgments

The author acknowledges the financial support by the Jimma University College of Medical and Health Sciences.

## Author contributions

A.M. conceived the idea for this study (primary investigator), participated in conception and design, in data collection, conducted data analysis, and drafted and finalized the manuscript for publication.

## Declaration of interests

The authors declare no competing interests.

## STAR★Methods

### Key resources table

**Table:** 

REAGENT or RESOURCE	SOURCE	IDENTIFIER
**Bacterial and virus strains**		

*Streptococcus pneumoniae*	American Type Culture Collection	ATCC 49619
*Staphylococcus aureus*	American Type Culture Collection	ATCC 25923
Streptococcus agalactiae	American Type Culture Collection	ATCC12386

**Biological samples**		

Vaginal swab	study participants	N/A

**Chemicals, peptides, and recombinant proteins**		

Todd-Hewitt broth	BD Diagnostics, USA	N/A
Amies transport media with charcoal	Oxoid, UK	N/A
Mueller–Hinton agar	Oxoid, UK	N/A
Blood agar	BD Diagnostics, USA	N/A
Christite, Atkins, Munch, Petersen(CAMP) tests	BD Diagnostics, USA	N/A
cotton swab	Medical wire and equipment, USA	N/A
penicillin G (10 IU)	Oxoid, UK	N/A
ampicillin (10 μg)	Oxoid, UK	N/A
clindamycin (2 μg)	Oxoid, UK	N/A
erythromycin (15 μg)	Oxoid, UK	N/A
Hippurate	BD Diagnostics, USA	N/A

**Deposited data**		

Raw and analyzed data	this paper	N/A

**Software and algorithms**		

software package for statistical data analysis	IBM Corp., Armonk, NY, USA	N/A

### Experimental model and study participant details

#### Study participants

In this cross-sectional study, a total of 200 pregnant women between 35 and 37 weeks of gestation attending routine antenatal care at Jimma University Hospital, Ethiopia, were enrolled from September 2019 to February 2020. Jimma is located 355 kilometers southwest of Addis Ababa, Ethiopia. Participation was voluntary, and informed written consent was obtained from all subjects after receiving ethical clearance from the Jimma University Ethics Review Board. Pregnant women who had taken antibiotics within two weeks prior to sample collection were excluded (*n* = 5). Clinical and socio-demographic information—including age, residence, education, occupation, gravidity, and previous obstetric history—was collected through a structured questionnaire administered by attending nurses. Vaginal swab specimens were collected following CDC guidelines for GBS screening^14^ and were processed at the microbiology laboratory within four hours of collection.

**Additionally**, the study did not assess or report sex or gender-related influences on GBS colonization, as all participants were pregnant women. Therefore, the findings cannot be generalized to non-pregnant populations or to individuals of other gender identities.

#### Quality control strains and identification protocols

To ensure laboratory quality control and methodological consistency, reference strains—*Streptococcus agalactiae* (ATCC 12386), *Streptococcus pneumoniae* (ATCC 49619), and *Staphylococcus aureus* (ATCC 25923)—were used to validate the performance of culture media, reagents, and antibiotic disks. GBS was identified using standard microbiological techniques including enrichment in selective Todd-Hewitt broth, subculture on 5% sheep blood agar, and confirmatory biochemical tests such as CAMP and hippurate hydrolysis.^34^

### Method details

#### Sample collection and transportation

According to the CDC guidelines,^14^ two vaginal swabs (one for direct gram stain and the other for inoculation) were collected by the attending nurse from the lower third of the vagina by inserting a sterile cotton swab (Medical wire and equipment, USA) 1–2 cm into the lower entrance of the vagina and by swabbing the sides of the vagina. Thereafter, the specimens were transported in Amies transport media with charcoal (Oxoid, UK) to the microbiology laboratory for culture within four hours of collection.

#### Isolation and identification of GBS

Vaginal swab was inoculated in Todd-Hewitt broth (BD Diagnostics, USA) containing nalidixic acid (15 mg/L) and gentamicin (8 mg/L) and incubated at 35°C–37°C in 5% CO_2_ for 24 h. Subculture was performed in 5% sheep blood agar (BD Diagnostics, USA) for isolation of GBS. Broth cultures showing no visible turbidity after overnight incubation was further re-incubated and then subcultured after 48 h on sheep blood agar. GBS identification was made by colony morphology, Gram stain, catalase reaction, hemolytic activity on sheep blood agar plates, hippurate (BD Diagnostics, USA) and CAMP (Christite, Atkins, Munch, Petersen) (BD Diagnostics, USA) tests.^34^

#### Antimicrobial susceptibility test

Antimicrobial susceptibility pattern of all GBS isolates was determined by Kirby–Bauer disk diffusion method on Mueller–Hinton agar (Oxoid, UK) supplemented with 5% sheep blood according to the guideline recommendations of the Clinical and Laboratory Standards Institute.^14^ The following antimicrobial discs and concentrations (in brackets) were used: penicillin G (10 IU), ampicillin (10 μg), clindamycin (2 μg), and erythromycin (15 μg) (all from Oxoid, UK). The plates were incubated at 37°Cs for overnight. The results were interpreted according to Clinical and Laboratory Standards Institute guidelines.^14^

### Quantification and statistical analysis

#### Degree of vaginal GBS colonization

For participants with positive vaginal culture results, the degree of GBS colonization was assessed quantitatively based on colony counts observed on blood agar plates.^7^ The degree of growth in the plates was recorded as follows.

**Table:** 

Degree of colonization	Colony count
+1	<10
+2	10–30
+3	31–50
+4	>50

+1 and +2 were taken light infection, and +3 and +4 were taken high infection.

#### Statistics

All statistical calculations were done using software package for statistical data analysis (SPSS) for windows version 20. Descriptive statistics were computed to determine the rate of GBS and other variables. The relationships between GBS colonization and various risk factors were tested using the Chi square test. A *p* value of ≤0.05 was considered indicative of a statistically significant.
